# Determinants of Patient-Perceived Primary Healthcare Quality in Lithuania

**DOI:** 10.3390/ijerph20064720

**Published:** 2023-03-07

**Authors:** Vaida Servetkienė, Roma Puronaitė, Birutė Mockevičienė, Karolis Ažukaitis, Danguolė Jankauskienė

**Affiliations:** 1 Health Research Laboratory, Mykolas Romeris University, 08303 Vilnius, Lithuania; 2 Vilnius University Hospital Santaros Klinikos, 08406 Vilnius, Lithuania; 3 Clinic of Pediatrics, Faculty of Medicine, Vilnius University, 03101 Vilnius, Lithuania

**Keywords:** healthcare accessibility, healthcare quality, patient experiences, patient-perceived healthcare quality, primary healthcare

## Abstract

Patient-centered care is considered to be one of the essential pillars of a modern healthcare system. Thus, quality assessment based on patients’ perceptions, views and experiences in their journey through the healthcare system is recognized as one of the key principles for quality improvement initiatives. Measuring patient satisfaction can be confounded by expectations and prior experiences, which can be at least partly overcome by evaluating patient-perceived healthcare quality (PPHQ). Understanding the principal constituents of PPHQ may aid healthcare professionals and decision makers in the healthcare management process and help in creating instruments to meaningfully measure patient feedback. Herein, we aimed to analyze the primary determinants of PPHQ and their interactions, with a focus on patient experiences and healthcare accessibility, using the example of Lithuanian primary healthcare. For this purpose, we conducted a cross-sectional representative telephone survey that included a total of 1033 respondents (48% male) who had encountered primary healthcare during last 3 years. Survey questions consisted of sociodemographic characteristics, patient perceptions of healthcare service provision, patient experiences, self-reported health status and overall PPHQ ranked with a 5-point Likert scale as the primary outcome. The classification-regression tree (CRT) technique was used to analyze the relationship between different explanatory variables and PPHQ, as well as their relative importance and interactions. The majority of respondents (89%) evaluated PPHQ as acceptable or good. CRT analysis identified staff behavior, organizational accessibility and financial accessibility as the most important factors affecting PPHQ. Importantly, the latter factors surpassed the effect of other known PPHQ determinants, such as sociodemographic characteristics or health status. Further analysis has revealed that the relative importance of staff behavior, including understanding, attention and empathy, increased when more problems with organizational accessibility were encountered. In conclusion, our study suggests that PPHQ in primary healthcare may primarily be determined by organizational and financial accessibility and staff behavior, which may also act as an important mediating factor.

## 1. Introduction

Patient-centeredness is considered as one of the essential elements of a well-functioning modern healthcare system. As such, patient-centered care (PCC) is recognized among the six domains of healthcare quality (HQ), according to the Institute of Medicine. PCC follows the principles of responsive healthcare, where the patient acts as the primary source of control [1,2,3]. Although one of the major goals of PCC is to improve health outcomes at an individual patient level, its benefits extend across the whole health service. These broader benefits may encompass the increased trust of the healthcare system within society, higher moral satisfaction among physicians and the increased cost-efficiency of healthcare services [4]. Finally, well-implemented PCC acts not only through improved patient–physician interactions but may also result in the decreased rates of avoidable hospitalizations, diagnostic procedures and medication prescriptions [5].

The successful implementation of PCC requires that certain principles be followed which allow the aforementioned control to be delegated the patient and thus requires the active involvement of patients in the healthcare service process. Davis et al. distinguish regular feedback from patients as one of the pathways for the successful implementation of PCC in the primary healthcare setting [3]. This feedback may include the collection of patient-reported outcome and experience measures (PROMs and PREMs, respectively), as well as measuring perceptions, views or overall satisfaction with delivered healthcare [6]. The latter is often assessed by conducting consumer satisfaction or patient opinion surveys in order to obtain aggregated information from the target group of individuals, shaped by their personal experiences, attitudes and perceptions. These are conducted systematically in certain countries, while in others they are limited to individual healthcare facility levels [7].

On the other hand, patients’ satisfaction conceptually reflects how healthcare service delivery meets their expectations and, hence, does not directly reflect higher quality. There are several reasons why the assessment of PPHQ (patient-perceived healthcare quality) may be superior to patient satisfaction for the improvement of HQ and PCC implementation. First and foremost, satisfaction by definition corresponds to the fulfilment of one’s expectations, needs and desires. Thus, it exhibits great inter-individual differences and dependence on past experiences and does not directly imply the higher quality of healthcare service. At best, being satisfied with a service may be considered to be an indicator of healthcare service adequacy but not high quality. Hence, high satisfaction is not equivalent to good HQ and, thus, the two terms should not be used interchangeably [8].

In contrast, PPHQ offers a more technical and broader understanding of a patient’s experience within the healthcare system. PPHQ can also be viewed as an interaction between patient expectations and experiences. On the other hand, the assessment of PPHQ may be seen as challenging due to its implicit nature and the unclear characterization of HQ among patients. PPHQ may reflect how healthcare service delivery meets patients’ needs, as opposed to solely their expectations [8,9,10].

Due to different interests, levels of maturity, common perceptions and knowledge of HQ among the different actors (stakeholders) involved in the healthcare system, HQ may be assessed from the perspective of patients, healthcare workers, researchers, managers and policy makers. For example, the current definitions of HQ used by the European Commission and the World Health Organization incorporate three principal dimensions: efficacy, safety and patient-centeredness. These definitions, largely shaped by healthcare researchers and policy makers, clearly identify responsiveness to patient needs as an essential component of HQ [11]. It is, however, unclear what domains and to what extent HQ is constituted from the perspectives of patients in different settings.

Thus, exploring patients’ understanding of key elements determining HQ may help to develop a fuller picture for HQ improvement initiatives. Conversely, patients’ perceptions of HQ may also shape their attitudes towards it, leading to (among other potential consequences) decreased trust and compliance.

In this context, it is important to acknowledge the importance of intersubjectivity, i.e., *what exists in the subjective consciousness of many individuals/a group* [12]. The identification of the intersubjective concepts of HQ determinants and their structure among patients is important for the development of strategies for the improvement of HQ. If the conditions or an individual’s characteristics change, leading to changes in the beliefs and attitudes of the majority of individuals, then the intersubjective phenomenon must also change. For example, changes in the most important determinants of PPHQ could likely modify PPHQ itself.

Given the potential benefits of PCC in healthcare decision making and the HQ improvement process, including the involvement of patients as active and informed participants, its implementation is of particular importance. This can be accomplished, at least in part, by assessing PPHQ and its primary determinants which, in conjunction with input from other stakeholders, can help to accommodate an understanding of HQ in individual healthcare settings.

PCC and the focus on patient involvement in the management and organization of HQ is becoming an increasingly important issue in Central and Eastern European (CEE) countries. However, compared to other Western countries, the CEE region is still largely underdeveloped in this area [7]. While the overall management of HQ in CEE countries has been improving rapidly in recent years, the factors that have the greatest impact on the HQ from the patient’s point of view remain a topical focus of analysis, allowing us to tailor quality improvement initiatives towards patients’ preferences.

Several prior studies have investigated the determinants of PPHQ in various healthcare settings [13,14,15,16,17,18,19,20,21]. However, the complex interactions of these factors and possible discrepancies due to different levels of PCC development and implementation between Western European and CEE countries are largely unknown. Thus, we conducted a representative national survey in Lithuania with the aim of identifying the key determinants of patient-perceived quality in primary healthcare and their interactions by applying a classification-regression tree (CRT).

## 2. Materials and Methods

### 2.1. Design and Setting

A cross-sectional representative telephone (CATI) survey was conducted by professional interviewers during March–April 2021 in Lithuania. Respondents were randomly selected from a list of individuals who had previously given consent to be contacted for population surveys. Respondents were considered eligible for participation if they were (i) citizens of Lithuania aged 18 years or older and (ii) had interacted with the primary healthcare system either personally or through a person they had been taking care of during past 3 years. The representativeness of the distribution of the survey sample by sex, age and place of residence was not less than 95% when compared to the data available from Statistics Lithuania for 2020.

In order to representatively address all potential healthcare recipients and to reflect their experiences as fully as possible, the respondents could rate either their own experience with healthcare services or that of the person they were taking care of (minors or other care recipients). The composition of the survey sample was based on the demographic characteristics of the healthcare recipients (i.e., the respondent or the respondent’s care recipient). Thus, all demographic information except for education, which was reported exclusively for the respondents, was collected for the healthcare recipient.

All data collection was completely anonymous. The quality of the interviews was ensured by the direct monitoring of at least 10% of calls. All survey respondents were informed about the aim of the survey and were free to opt out of participation.

### 2.2. Survey Content and Variable Selection

This survey was one component of a project intended to develop an original PREM (patient-reported experience measures) tool to assess and identify patients’ experiences during their journey through the healthcare system. The questionnaire was created by a group of experts consisting of healthcare specialists, sociologists and healthcare managers based on the European Primer on Customer Satisfaction Management [22] and Lithuanian guidelines for evaluating customer satisfaction with public services [23]. Content was further tailored based on the analysis of a previously conducted focus group involving patients and their family members which aimed to explore patients’ experiences on their journey through the Lithuanian healthcare system [24]. The part of questionnaire concerning primary healthcare, in addition to the questions related to sociodemographic characteristics, consisted of perceptions of different elements of primary healthcare provision, patient experiences (frequency of encountering issues in the healthcare system) and overall PPHQ. Given the close link between healthcare service usage and a person’s health status, we also included a question about self-reported overall health status as a potential explanatory variable. The primary outcome of interest in this analysis was overall PPHQ (rated on a 5-point Likert scale, from *very poor* to *very good*). All other questions were considered explanatory variables and included the following domains:

- *Sociodemographic characteristics:* age; gender; place of residence; type of household; income; education; employment; and insurance by national health coverage.
- *Perceptions on different elements of primary healthcare service provision:* geographic accessibility; organizational accessibility; financial accessibility; information accessibility; perceived competence of physicians; behavior of medical staff; and infrastructure and facilities (5-point Likert scale from *very poor* to *very good*);
- *Patient experiences in terms of frequency of encountering issues with healthcare service provision:* difficulties in accessing primary healthcare (e.g., appointment scheduling); lack of empathy from medical staff; perceived insufficiency of diagnostics; lack of information from medical staff; difficult reimbursement procedures; and lack of interprofessional collaboration (5-point Likert scale, from *never* to *always*).
- *Self-reported overall health status* of the respondent (or care recipient) rated on a 5-point Likert scale, from *very poor* to *very good*.

Details on the specific formulations of the questions are provided in Table A1. The group of questions “*Perceptions on different elements of primary healthcare service provision*” (Table A1, questions 1–7) was constructed to reflect the overall assessment of the dimension based on patients’ personal experiences. The second group of questions, namely “*Patient experiences in terms of frequency of encountering issues with healthcare service provision*” (Table A1, questions 8–13), captured the actual frequency of encountering a certain experience.

The Cronbach’s alpha value for patients’ perceptions on the accessibility and quality of care was 0.91, indicating high internal consistency.

### 2.3. Analysis

Respondent characteristics were described by frequencies according to predefined categories. Responses to other survey questions that were all designed with 5-point Likert scale answers were also described as frequencies.

The associations of explanatory variables with the primary outcome (PPHQ) was analyzed using the classification-regression tree (CRT) method. Similarly to traditional methods, such as linear or logistic regression modelling, CRT allows the association between a response variable and multiple explanatory variables to be investigated. However, in contrast to conventional regressions models, a non-parametric test CRT allows interactions between multiple explanatory variables to be explored by identifying homogenous subgroups of the sample that exhibit the most differences with respect to the primary outcome. Being less sensitive to multicollinearity, CRT allows not only the direction and strength of the association effect to be investigated but also its hierarchical interaction with other variables.

Depending on the nature of the response variable (linear, ordinal or categorical), the CRT method finds explanatory variables by which the population varies the most in terms of the outcome. This variable constitutes the parent node, which further branches into binary child nodes. These child nodes are split further depending on other explanatory variables, which are the strongest predictors of the largest subgroup differences. Eventually, the generated CRT tree terminates based on the rules that are presented, further resulting in different subgroups of patients that differ regarding the primary outcome. Thus, in the simplest terms, CRT acts as a decision tree, aiding the prediction of the response variable based on selected explanatory variables. A detailed description of the CRT method and its applicability have been published elsewhere [25].

The following rules were applied for the CRT used in this analysis to avoid overfitting the model and preventing it from becoming too complex and difficult to interpret:

- maximum number of levels: 4;
- minimum sample size for parent nodes: 10% of the sample;
- minimum sample size for child nodes: 1% of the sample.

First, questionnaire responses and demographic characteristics were provided as explanatory variables and PPHQ as response variable for the CRT algorithm. Applying predefined conditions to the depth of the tree and the sample size in the nodes for the first model, but without any assumptions for the variable at first branch, the CRT algorithm selected the variable whose responses the most appropriately divided the sample into heterogenous, but internally homogenous, groups according to the response variable (PPHQ). Using the same principle, all nodes were subdivided into smaller ones, choosing from all explanatory variables at each step, until no more splitting was achieved, or the model constraints were met.

The primary CRT model was built with the explanatory variable resulting in the best prediction improvement as the primary splitting variable (CRT model 0). Additionally, two other models with forced primary splitting variables that showed comparably high prediction characteristics were built to explore possible changes in interactions.

The CRT algorithm handles missing values using surrogate splits. The decision regarding which child node to choose for the variable with missing values was made by classifying observations according to the solution of the most similar variable as a surrogate variable for the split.

The importance of the variables based on their ability to find the most different subgroups of the population was assessed by calculating the relative importance score of each explanatory variable. The uncertainty of CRT trees was assessed by calculating classification error using 10-fold cross-validation, which corresponded to the misclassification rates after randomly removing certain subsets of the sample.

All statistical analyses were performed using SPSS package 29.

## 3. Results

### 3.1. Sample Description

The sample consisted of 1033 respondents (47.6% male), who were sampled in a way that proportionally reflected the distribution of the Lithuanian population by place of residence, gender and age groups. The distribution (proportions) of the sample by age groups and gender was consistent with the proportion of the Lithuanian population in 2020, according to the data of the Department of Statistics, by at least 95%, and of the population by place of residence by at least 97%. The proportion of respondents who cared for other healthcare recipients (minors) in the total sample was 17.1%. A total of 33% of respondents had higher education and most were employed (65%) and had insurance via national health coverage (95%). Further details on respondent characteristics are provided in Table 1.

### 3.2. General Data on PPHQ in Primary Healthcare (Descriptive Analysis)

**Figure 1:**
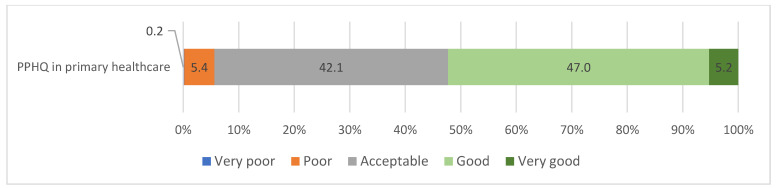
Distribution of responses regarding PPHQ in primary healthcare.

The majority of respondents evaluated PPHQ as acceptable (42.1%) or good (47%), while only two respondents (0.2%) evaluated PPHQ as very poor (Figure 1).

**Figure 2:**
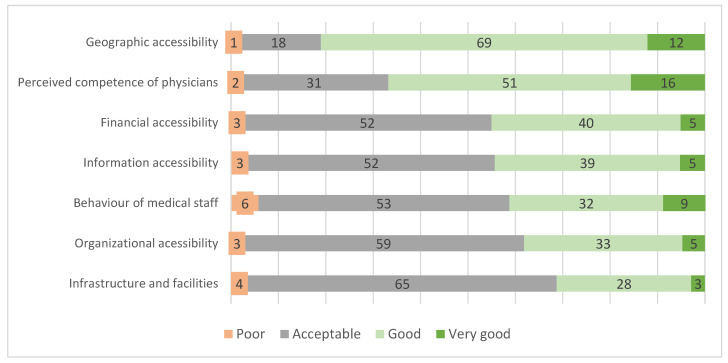
Assessments of determinants of PPHQ in primary healthcare (in percent of respondents, *n* = 1033).

None of the respondents evaluated any of the domains related to accessibility and quality of services as very poor. Nevertheless, a number of current problems were indicated. Geographical accessibility and the perceived competence of physicians were among the highest-rated domains, with 81% and 67% of the respondents rating them very good or good. All remaining domains were predominantly rated as acceptable, with financial and information accessibility and the behavior of medical staff rated comparably. The organizational accessibility and infrastructure/facilities domains received the worst ratings. Detailed data on these ratings are presented in Figure 2.

It is of note that over 60% of the respondents aged over 65 years rated the behavior of the medical staff towards them as very good or good, in comparison to only 21% of the respondents aged 35–44 years (*p* < 0.001). Other sociodemographic characteristics were not associated with other survey responses.

**Figure 3:**
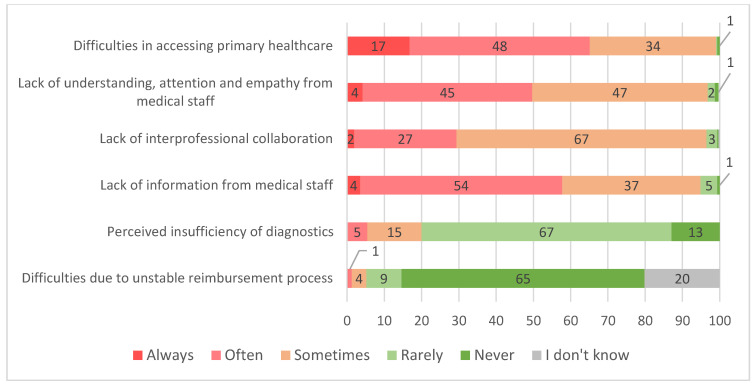
Frequency (based on experience) of patients encountering problems (in percent of respondents, who reported having experienced difficulties, *n* = 308).

Overall, 29.8% of the respondents reported encountering difficulties with the healthcare system. Of those, the most frequent were difficulties in accessing primary healthcare (e.g., appointment scheduling) and lack of understanding, attention and empathy from staff. These two areas were encountered at least “often” in 65% and 50% of cases, respectively. A lack of interprofessional collaboration and information from medical staff were encountered at least “sometimes” by the majority of respondents, while perceived insufficiency of diagnostics and difficulties in reimbursing procedures were generally encountered “rarely” (Figure 3).

### 3.3. Main Determinants of PPHQ in Primary Healthcare (CRT Analysis)

#### 3.3.1. Primary CRT Model

Despite the fact that the initial analysis of the survey data provides some indication of which factors were most positively or negatively assessed by the respondents, and that their perceptions were strongly influenced by their experience in assessing/using primary healthcare services, the CRT analysis provides additional in-depth insights into the influence of certain determinants on PPHQ in primary healthcare and their interaction.

**Figure A1:**
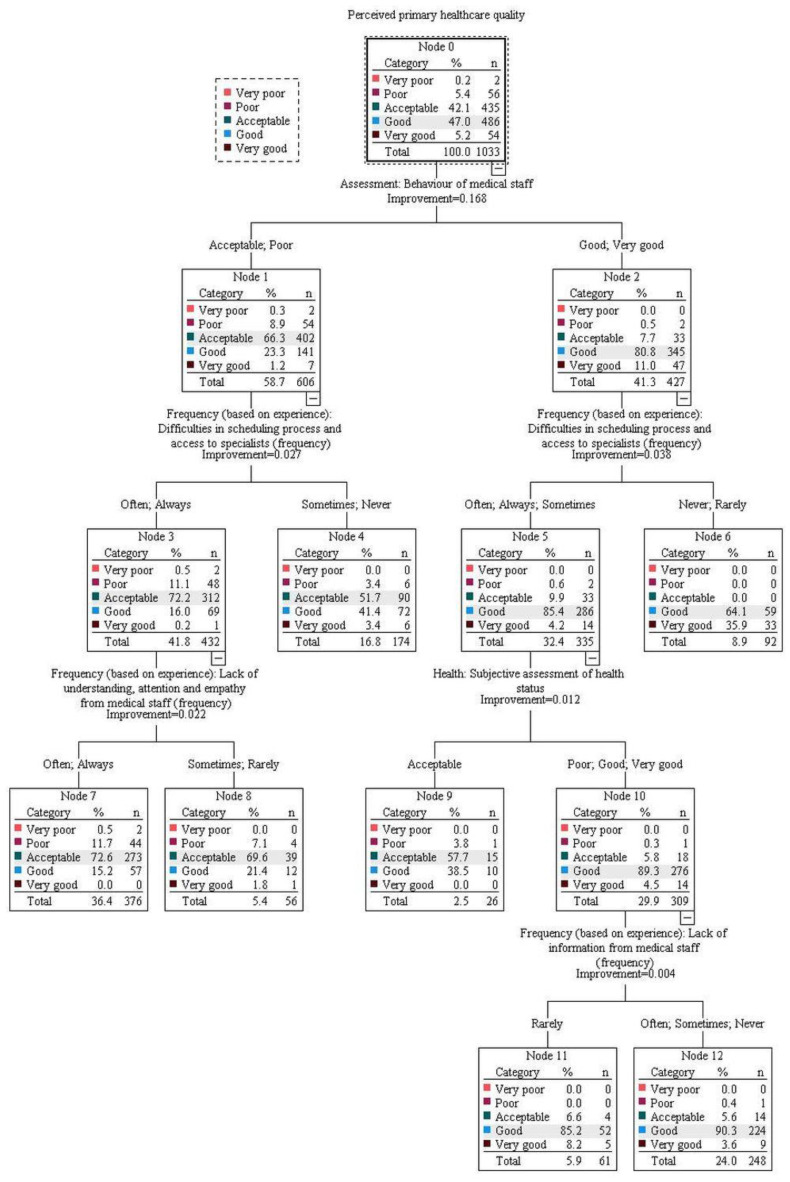
CRT model 0.

Based on the primary model, selected due to its higher discrimination capacity, the most important factor determining PPHQ was the behavior of medical staff (CRT model 0; Figure A1). Respondents who evaluated staff behavior as good or very good had more positive perceptions of PPHQ (Node 2). Among this group of respondents, the next most important factor was the frequency of difficulties in the scheduling process and access to primary healthcare physicians: those who experienced these problems at least “sometimes” had a more negative perception of PPHQ (Node 5). Further perceptions in this group were most strongly influenced by the subjective assessment of health status, with those evaluating the health of themselves or persons they care for as anything other than “acceptable”, perceiving PPHQ as better (Node 10). The final split in this group of respondents was observed based on the frequency of lack of information from medical staff (Nodes 11 and 12).

When analyzing those who had initially worse perceptions of PPHQ based on the behavior of staff evaluated as being acceptable or poor (Node 1), the frequency of difficulties in the scheduling process and access to specialists was the second most important determining factor. However, in this group of patients, those who were experiencing these problems more frequently (Node 3) had a further split in their perceptions on PPHQ based on the frequency of experiencing a lack of understanding, attention and empathy from medical staff. Respondents who experienced these issues at least “often” had the most negative perceptions of PPHQ (Node 7).

#### 3.3.2. CRT Models with Substituted Primary Splitting Variable

We further constructed two additional CRTs by forcing variables with the highest relative importance and classification improvement scores as primary splitting variables: organizational accessibility (CRT 1; importance score 0.181 and improvement parameter 0.163) and financial accessibility (CRT 2; importance score 0.175 and improvement parameter 0.146) (Table 2).

**Figure A2:**
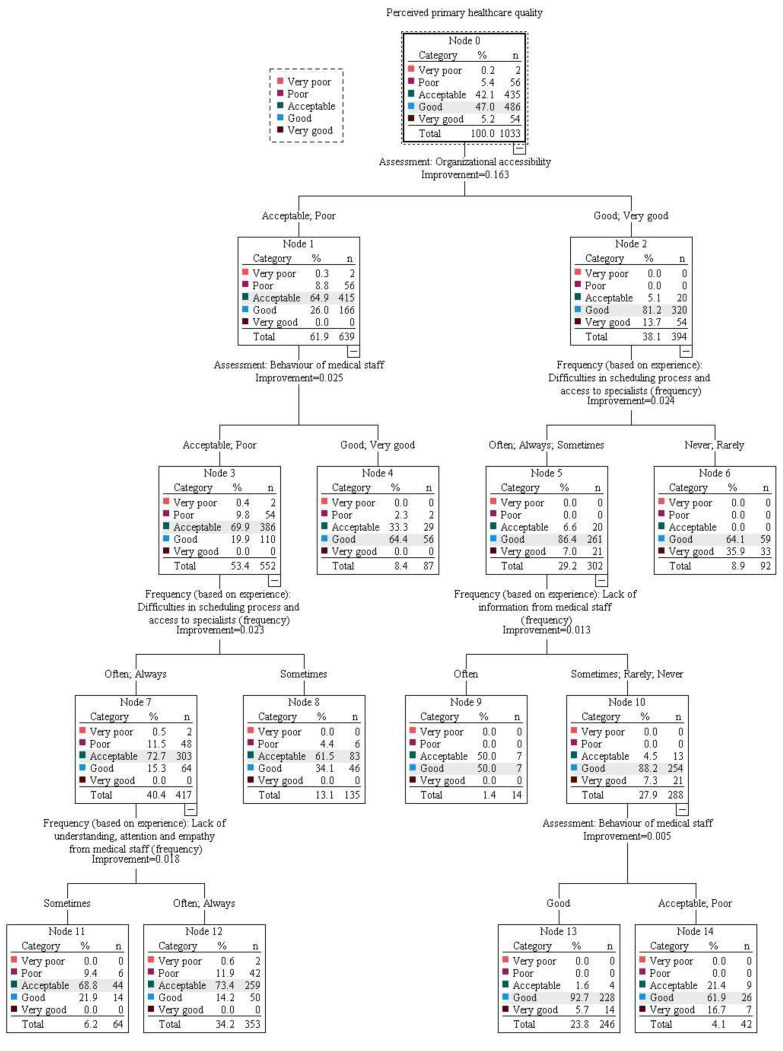
CRT model 1.

In the model with organizational accessibility forced as the primary variable (CRT model 1; Figure A2), among those who evaluated it as good or very good (Node 2), further evaluations were influenced most strongly by the frequency of encountering problems in this area. Those experiencing difficulties in scheduling and access to specialists more often (Node 5) had more negative perceptions and were divided further based on lack of information from medical staff. Respondents experiencing a lack of information less frequently (Node 10) had better perceptions of PPHQ in primary healthcare and were divided further based on the behavior of medical staff. The largest proportion of respondents evaluating PPHQ as at least “good” was observed in those who assessed behavior as “good” (Node 13).

Among those in CRT 1 who evaluated organizational accessibility more poorly (Node 1), the behavior of medical staff was the next most important dividing variable. More negative perceptions of PPHQ were observed among those who evaluated the behavior of medical staff as “acceptable” or “poor” (Node 3). In this group of respondents, further evaluations of PPHQ were then influenced by the frequency of encountering problems in healthcare organization—with more frequent encounters leading to more negative perceptions (Node 7). The final dividing factor in the latter group was the frequency of lack of understanding, attention and empathy from staff, resulting in the most negative evaluations among those experiencing these problems at least often (Node 12).

**Figure A3:**
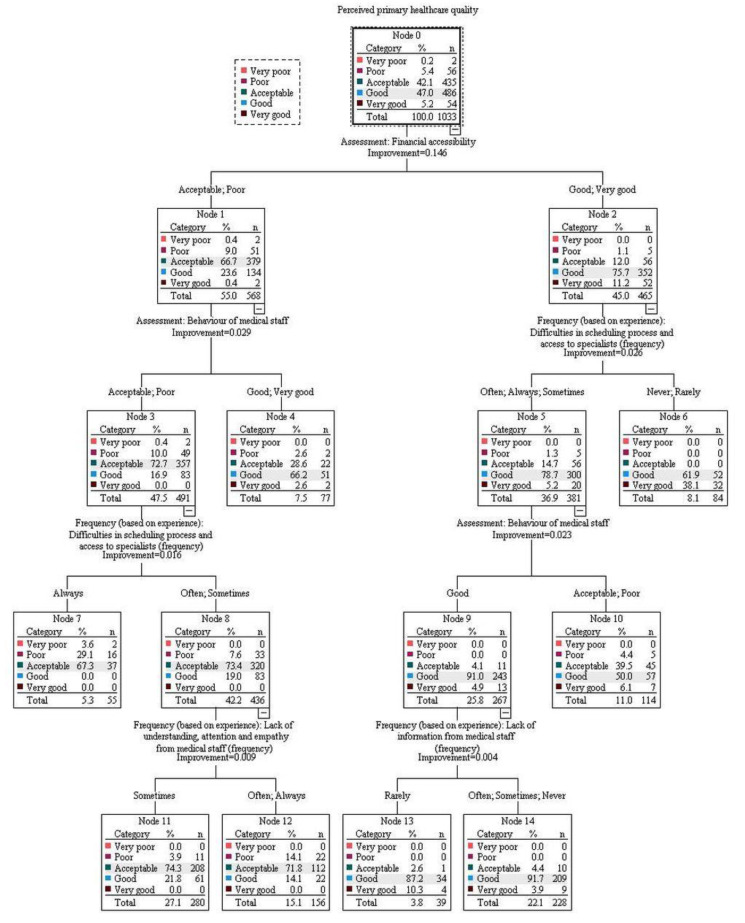
CRT model 2.

In the CRT model 2 (Figure A3), more positive perceptions of PPHQ were observed by those evaluating financial accessibility as “good” or “very good” (Node 2). Among those respondents, the evaluation of PPHQ was further influenced by the frequency of encountering problems with scheduling and access to specialists with more frequent encounters, resulting in less positive perceptions (Node 5). In this group of respondents, the next most important factor was the behavior of medical staff, with best perception among those evaluating it as “good” (Node 9). The final split in this group of respondents was observed based on frequency of a lack of information from medical staff (Nodes 11 and 12).

In the group of respondents who evaluated financial accessibility more negatively, resulting in more negative perceptions of PPHQ (Node 1), the second most important factor was the behavior of medical staff. More negative perceptions were observed in those who evaluated behavior as acceptable or poor (Node 3) and this was further influenced by the frequency of encountering problems with scheduling and access to specialists. If respondents encountered these problems at least often, they had more negative perceptions of PPHQ (Node 8). The latter group was then further split based on the frequency of the lack of understanding, attention and empathy from medical staff, with the most negative perceptions in those experiencing these issues more frequently (Node 12).

All three models had comparable classification errors in cross-validation analysis: 0.28, 0.26 and 0.26 for CRT 0, 1 and 2, respectively.

## 4. Discussion

In the present study, we explored the most important determinants of PPHQ in the primary healthcare setting in Lithuania as a representative country of the CEE region. In order to understand the complexity of decision pathways leading to differing perceptions of PPHQ, we applied CRT as an alternative method to traditional regression models. Our analysis revealed that the most important determinants of PPHQ in the primary healthcare setting are the behavior of medical staff, organizational accessibility and financial accessibility. Importantly, our analysis has shown that these factors each appear to act as mediators of one another for PPHQ evaluation, with staff behavior acting as the most important positive compensating factor. The influence of other known factors, such as sociodemographic characteristics or subjective health status assessment, appear to be attenuated and relatively insignificant compared to the influence of healthcare service accessibility and actual experiences.

Although the nature of our study only allows the importance of dimensions that were actually evaluated in our survey to be determined, their composition is comparable to those used in other HQ studies. A previous qualitative study by Levine et al. of patients and physicians reported physicians’ clinical skill, rapport (physician–patient relationships) and health-related communication as common primary elements of quality perceptions in ambulatory care [13]. This is reiterated in other studies that identified communication and information, accessibility, courtesy and emotional support [14], infrastructure and assurance [15,16], empathy [14], patient-centeredness, technical skills [14,17,18,19,20], waiting time [20,21] and urgency [20,21] as important determinants of HQ from the perspective of the patient.

### 4.1. The main Determinants of Healthcare Quality from Patients’ Perspectives

#### 4.1.1. The Behavior of Medical Staff Factor and Its Interaction with other PPHQ Determinants

In our study, positive behavior of medical staff was described as interactions with the patient in an attentive, respectful, polite, empathetic and responsible manner, recognizing the patient’s individual needs. The strong association between PPHQ and different dimensions of medical staff behavior has already been reported in other studies [14,17]. According to Patey et al., healthcare professional’s behavior serves at least six different purposes, which also includes building therapeutic alliances with care receivers through collaboration, communication and empathy with respect. If achieved successfully, this leads to the better engagement of the patient in the healthcare process. For example, this may motivate the patient to seek medical attention in the event of recurrent health problems, to be compliant with the agreed treatment plan and to cooperate in the management of health conditions [26,27].

Notably, we have observed that the behavior of medical staff appears to be more important for those patients who face greater organizational or financial difficulties during their journey in the healthcare system. From the healthcare management perspective, it is important to acknowledge that for patients experiencing fewer interruptions (organizational or financial) during their journey, the behavior of medical staff becomes less important when evaluating PPHQ. This means that when patients face limitations on accessibility (difficult registration, long waiting time), then attention and empathy shown to the patient, as quality indicators, can significantly increase the positive assessment of HQ. Similar trends were reported by Etingen et al.—those patients who were provided with adequate preventive care services less frequently prioritized such factors as empathy, holistic care, participation and respect as necessary elements of the service [28].

#### 4.1.2. Organizational Accessibility and Its Interaction with Other PPHQ Determinants

Examining patients’ perceptions of administrative processes in healthcare facilities is an integral part of understanding patients’ perceptions of HQ. We included different components of these processes under the assessment of organizational accessibility, which included length of opening hours, appointment procedures, queuing, waiting times, immediate responses and problem-solving speed. It was noted that patients felt more convenience and satisfaction with their treatment if access to services was improved [29,30]. Studies in various healthcare service sectors have shown that, in the area of organizational accessibility, customers’ or patients’ opinions are most often affected by delays that they perceive to be unreasonable, and this could cause dissatisfaction, anger or frustration [30,31].

In line with these notions, our CRT analysis also confirmed that organizational accessibility, which includes the speed of service delivery (length of opening hours, appointment procedures, queuing, waiting times; whether patient appointments run at the scheduled time; whether there is an immediate response/problem-solving) is one of the key determinants of PPHQ. As mentioned previously, the importance of organizational accessibility for overall PPHQ assessment is strongly related to the behavior of medical staff. Those who evaluate organizational accessibility as “good” place less priority on information accessibility, highlighting the importance of addressing and regularly monitoring organizational issues.

Notably, factors such as waiting time (which was included in our study as an integral part of organizational accessibility) appear to be more important in developing countries. The significance of this factor prevails in regions where services, resources and administrative coordination are often not well developed [32,33]. Results from other studies reveal that waiting times had the largest perceived difference between patients’ perceptions and expectations, while physicians’ competence showed the smallest difference between expected and perceived ratings [34].

The results of our CRT analysis are also close to the aforementioned previous studies, highlighting the importance of organizational accessibility and speed of service delivery, and the behavior of medical staff, including empathy, on the overall patient-perceived HQ score.

#### 4.1.3. Financial Accessibility and Its Interaction with Other PPHQ Determinants

In our study, respondents evaluated the financial accessibility perspective regarding their own financial capacity in terms of financial affordability (if the service was fully or partially paid-for), whether the price of the service was financially affordable to the patient and through a cost–efficiency perspective.

Financial accessibility was identified as being among the most important determinants of PPHQ, despite the fact that the absolute majority of survey respondents had full national health insurance. With financial accessibility, we observed similar trends as previously. The worse the financial accessibility is rated, the more important the behavior of medical staff becomes. If financial accessibility is rated better, then organizational issues and information accessibility become more important.

These results are similar to those of other studies. According to Reinhardt et al., private healthcare costs correlate negatively with patient satisfaction: an increase in private healthcare costs leads to a 98.7% decrease in patient satisfaction [35]. This relationship and the perceptions of patients seem perfectly reasonable, since citizens, although contributing to public healthcare costs through taxes, have to pay extra to receive private healthcare which is faster or of better quality when public healthcare services do not guarantee this. As noted in another study, negative patient assessment may also be due to perceived unfairness: patients may believe that the distribution of the financial burden of financing is unfair, even though the healthcare system is functioning well [36].

The financial affordability of healthcare services has also been noted by other researchers as an important factor influencing the meeting of patients’ medical needs and their satisfaction with HQ [37]. EU-SILC data for 30 European countries also confirm that lower household purchasing power creates financial barriers to accessing healthcare, as well as indirect costs for any income quintile, in terms of waiting time, travel costs and time spent not working [38].

#### 4.1.4. Self-Reported Overall Health Status and Sociodemographic Characteristics

Characteristics such as age, sex, health status and pain have long been known to have significant relationships with the assessment of the quality of healthcare services. Sociodemographic characteristics and health state are recognized as principal components in the conceptual model of patient satisfaction with primary care [39]. A recent study also suggested that differences in the quality and accessibility of healthcare are due both to the changing needs of patients over their lifetime (at different age stages) and to their socio-economic status, i.e., identifying differences between employed and non-employed patient groups [40]. Although there were differences in the assessment of the behavior of healthcare staff depending on age (older subjects having more favorable evaluations), CRT analysis revealed that sociodemographic characteristics such as age, gender and employment status were of relatively low importance for PPHQ. This finding, deviating from those previously reported by other groups, suggests that accessibility and experiences surpass the effects of other non-modifiable characteristics. Self-reported overall health status only influenced the evaluation of those who had generally better experiences of health service providers’ behavior but experienced more problems regarding organizational accessibility.

### 4.2. CRT as a Method to Identify Patients’ Individual Needs for Targeted HQ Improvement

Considering the aforementioned challenges of interpreting and analyzing patient perceptions, traditional analysis methods, such as linear regression models, may be insufficient to address the heterogeneity of the patient population and the complexity of interactions between different elements related to HQ. Thus, we employed CRT as a novel method of studying HQ that allows the association between a response variable and multiple explanatory variables to be investigated by identifying homogenous subgroups of the sample that exhibit the most differences with respect to the primary outcome. In our study, the CRT method allowed us to identify certain homogeneous groups of patients according to preferences and mindsets specific to Lithuania, as well as the hierarchical interaction of variables.

### 4.3. Implication of the Study

As many international studies [8,30,36,37,41] show alongside this contribution, a number of factors may influence the quality assessment of the provision (as a process) of healthcare services. These factors may also have different effects on the overall assessment of HQ or on the performance of a particular facility, due to the relevance of each factor being perceived differently by each individual. Therefore, a more detailed analysis is needed for both healthcare decision makers and healthcare facility managers in order to be able to carry out an informed assessment of the effectiveness of the services provided to a patient, the smoothness of these processes and the sufficiency of resources. This will enable decisions to be made regarding the improvement of the services needed by patients and will ensure that patients’ needs are met.

The determinants identified in our study are valuable for the development and implementation of original and country or region-specific PREMs. Based on the results of this study, it is important to emphasize that the development of country- or disease-specific PREMs should include, as key determinants, the behavior of medical staff; organizational accessibility, including access to primary healthcare and waiting times; and patients’ perception of financial accessibility and the affordability of healthcare services.

### 4.4. Limitations of the Study

This study has several limitations. First, the survey was conducted one year after the COVID-19 pandemic began, i.e., when certain healthcare services were already being delivered remotely, and this may have influenced the results of the assessments of the quality and accessibility of healthcare services. However, we aimed to address this issue by asking respondents to refrain from including issues related specifically to the COVID-19 pandemic when answering survey questions.

Second, quantitative research through surveys alone, especially when close-ended questions are used, cannot always provide comprehensive data on patients’ perceptions of HQ. Therefore, in order to more precisely identify the determinants of patient-perceived HQ and relevant interactions, it would be appropriate to conduct additional qualitative research, either through interviews or focus group discussions with both patients and healthcare providers.

Thirdly, in order to minimize public health risks posed by the COVID-19 pandemic, the survey was conducted by telephone interviewing (CATI) rather than face-to-face (FtF), thus it must be taken into account that the CATI survey method may also carry a risk of reducing representativeness by excluding individuals who do not use phones.

Finally, we were only able to evaluate the importance of domains that were included in our survey. Although the survey was developed based on known tools used in healthcare management, expert opinions and the results of focus groups involving patients, other aspects that were not included in the scope of our survey could affect the assessment of PPHQ.

## 5. Conclusions

Our representative national survey showed that every third respondent encountered problems in their journey through the primary healthcare system in Lithuania. Despite this, the majority of respondents rated PPHQ as “acceptable” or “good” overall. The application of CRT as a novel method to explore the determinants of PPHQ and their interplay identified healthcare staff behavior, organizational accessibility and financial accessibility as the primary determinants of PPHQ in primary healthcare services. Importantly, healthcare staff behavior may act as a modifier of PPHQ assessment in instances where other aspects of healthcare provision are evaluated as inadequate, leading to more positive healthcare assessments. Thus, these issues should be considered among priorities for HQ improvement initiatives and should be included in tools for measuring patient experiences in the CEE region.

## Figures and Tables

**Table 1:** Sociodemographic characteristics of respondents.

Sociodemographic Characteristics	Number of Respondents	Proportion (Proportions of the Lithuanian Population in 2020 from Official Statistics)
**Age group**		
0–17 years	177	17.1% (20%)
18–24 years	66	6.4% (6%)
25–34 years	155	15.0% (13%)
35–44 years	140	13.6% (12%)
45–54 years	166	16.1% (14%)
55–64 years	144	13.9% (15%)
≥65 years	185	17.9% (20%)
**Male**	**492**	**47.6% (47%)**
**Female**	**541**	**52.4% (53%)**
**Place of residence**		
Town (up to 5000 inhabitants)	318	30.8% (30%)
City (5000–80,000 inhabitants)	218	21.1% (22%)
City (>80,000 inhabitants)	497	48.1% (48%)
**Education**		
Higher	343	33.2%
Primary or lower secondary	27	2.6%
Vocational	295	28.6%
Secondary education	368	35.6%
**Employment status**		
Unemployed	76	7.4%
Employed (private sector)	531	51.4%
Employed (public sector)	155	15.0%
Retired	115	11.1%
Student	152	14.7%
Did not indicate	4	0.4%
**Income (per household member)**		
<EUR 400	68	6.6%
EUR 401–EUR 600	347	33.6%
EUR 601–EUR 800	474	45.9%
>EUR 800	79	7.6%
Did not indicate	65	6.3%
**Type of household**		
One person	113	10.9%
One adult with children	44	4.3%
Two adults with children	250	24.2%
Two adults, at least one aged 65 or over, without children	40	3.9%
Two adults aged under 65 without children	502	48.6%
Three or more adults without children	84	8.1%
**Insured by national health coverage**	**977**	**94.6%**

**Table 2:** The relative importance of explanatory variables.

Independent Variable	Importance	Normalized Importance
Organizational accessibility	0.181	100.0%
Behavior of medical staff	0.178	98.4%
Financial accessibility	0.175	96.3%
Information accessibility	0.156	86.1%
Lack of understanding, attention and empathy from medical staff (frequency)	0.096	52.7%
Difficulties in accessing primary healthcare (frequency)	0.093	51.5%
Infrastructure and facilities	0.087	47.9%
Perceived competence of physicians	0.058	32.1%
Perceived insufficiency of diagnostics (frequency)	0.054	29.7%
Lack of information from medical staff (frequency)	0.047	25.7%
Geographic accessibility	0.045	24.9%
Subjective assessment of health status	0.044	24.2%
Type of household	0.030	16.4%
Place of residence	0.029	15.9%
Lack of interprofessional collaboration (frequency)	0.023	12.6%
Age	0.013	7.1%
Employment status	0.010	5.7%
Income status	0.009	5.2%
Difficulties due to unstable reimbursement process (frequency)	0.006	3.3%
Education status	0.002	1.3%
Insurance by national health coverage status	0.002	1.2%

## Data Availability

The data presented in this study are available from the authors on request.

## Appendix A

**Table A1:** Determinants of the overall assessment of PPHQ in primary healthcare, as selected during the survey.

I. Assessment of the PPHQ of primary healthcare based on patients’ personal experiences Question to respondents: How do you feel about the following determinants when you refer to your primary healthcare facility and receive services? Please rate each of the determinants according to your personal experience (5-point Likert scale, from *very poor* to *very good*)		
No.	Determinants	Question and description of the determinant given to the respondent during the survey
1.	Geographic accessibility	Is the HC facility easily accessible and conveniently located? (i.e., availability of public transport, well-developed road network, car parking, etc.)
2.	Organizational accessibility	How do you rate the way in which the HC facility organizes service provision? (the length of opening hours, appointment procedures, queuing, and waiting times; whether patient appointments run at the scheduled time; whether there is an immediate response/problem-solving such as referral for tests, issue of necessary prescriptions; whether the patient is passed from one physician to another)
3.	Financial accessibility	How do you rate the financial accessibility of HCSs? (if a service is fully or partially paid for, does the patient think that the price of the service is financially accessible, is the price/quality ratio optimal?)
4.	Information accessibility	How do you rate the accessibility of information and provision of patients with information? (is it easy to find/obtain accurate, complete and comprehensible information? Are patients informed about the process of medical treatment, results, etc.?)
5.	Perceived competence of physicians	How do you rate the competence of physicians of the healthcare system? (are the professionals knowledgeable about their work?)
6.	Behavior of the medical staff	How do you rate the behavior of the medical staff towards patients? (i.e., are they respectful, polite, empathetic, responsible? Is the physician able to recognize/listen to the patient’s needs?)
7.	Infrastructure and facilities	How do you rate the physical environment of the healthcare system? (are the premises clean and tidy, with the necessary infrastructure, medical diagnostic equipment, supplies to provide the service, etc.?)
II. Patient experiences in terms of frequency of encountering the following issues Question to respondents: Have you encountered issues with healthcare services? If *yes*, how often have you encountered the following issues? (5-point Likert scale, from *never* to *always*)		
No.	Determinants	Question and description of the determinant given to the respondent during the survey
8.	Difficulties in accessing primary healthcare (e.g., appointment scheduling)	How often have you encountered difficulties with appointment and access to a primary care physician (including general practitioner (GP), pediatrician, obstetrician–gynecologist, surgeon)?
9.	Lack of empathy from medical staff	Is there a lack of understanding, attention and empathy from the medical staff?
10.	Perceived insufficiency of diagnostics	Do you think that the tests and comprehensive diagnostics ordered by physicians are sufficient?
11.	Lack of information from medical staff	Is there a lack of information from the medical staff about the illness, medical treatment and procedures?
12.	Difficulties due to unstable reimbursement process	Do you encounter issues with unclear, constantly changing procedures for reimbursement of medicines or medical supplies?
13.	Lack of interprofessional collaboration	Do you think there is a lack of collaboration between the professionals of different healthcare facilities (information sharing, trust, complementarity of one another’s actions)? For example, does your GP collaborate with specialist physicians when necessary?
